# Bedroom media, sedentary time and screen-time in children: a longitudinal analysis

**DOI:** 10.1186/1479-5868-10-137

**Published:** 2013-12-17

**Authors:** Andrew J Atkin, Kirsten Corder, Esther M F van Sluijs

**Affiliations:** 1 UKCRC Centre for Diet and Activity Research (CEDAR), MRC Epidemiology Unit, University of Cambridge School of Clinical Medicine, Institute of Metabolic Science, Cambridge Biomedical Campus, Box 285, Cambridge CB2 0QQ, UK; 2 MRC Epidemiology Unit, University of Cambridge School of Clinical Medicine, Cambridge, UK

**Keywords:** Television, Children, Family, Sedentary behaviour, Accelerometer

## Abstract

**Background:**

Having electronic media in the bedroom is cross-sectionally associated with greater screen-time in children, but few longitudinal studies exist. The aim of this study was to describe longitudinal patterns of ownership and examine cross-sectional and longitudinal associations of bedroom media with children’s sedentary behaviour.

**Methods:**

Data are from the Sport, Physical activity and Eating behaviour: Environmental Determinants in Young people (SPEEDY) study, collected at 3 time-points: baseline (2007, T_0;_ age 10.3 ± 0.3 years), 1-year (T_1y_) and 4-year (T_4y_) follow-up. For each assessment, 1512 (44.9% male), 715 (41.0% male), and 319 (48.3% male) participants provided valid accelerometer data. Outcome variables were accelerometer-assessed sedentary time and self-reported screen-time. The presence of a television or computer in the bedroom was self-reported by participants and a combined bedroom media score calculated as the sum of such items. Cross-sectional and longitudinal associations between bedroom media and each outcome were examined using multi-level linear regression.

**Results:**

Bedroom TV ownership fell from 70.9% at T_0_ to 42.5% at T_4y_. Having a TV in the bedroom (beta; 95% CI*100, T_0_: -1.17; -1.88, -0.46. T_1y_: -1.68; -2.67, -0.70) and combined bedroom media (T_0_: -0.76; -1.26, -0.27. T_1y_: -0.79; -1.51, -0.07) were negatively associated with objectively measured weekly sedentary time at T_0_ and T_1y_. Having a computer in the bedroom (beta; 95% CI, T_0_: 0.15; 0.02, 0.29. T_4y_: 0.35; 0.10, 0.60) and combined bedroom media (T_0_: 0.09: 0.01, 0.18. T_4y_: 0.20; 0.05, 0.34) were positively associated with screen-time at T_0_ and T_4y_. Relative to participants without a computer throughout the study, children that had a computer in their bedroom at T_0_ but not at T_4y_ (beta; 95% CI for change in screen-time: -8.02; -12.75, -3.29) reported smaller increases in screen-time.

**Conclusions:**

The bedroom media environment changes with age and exhibits a complex relationship with children’s sedentary behaviour. Modifying children’s bedroom media environment may impact upon screen-time but appears unlikely to influence overall sedentary time.

## Introduction

Sedentary behaviours, such as watching television (TV) and using a computer, are highly prevalent during childhood [1-4] and may be adversely associated with cardiometabolic health, though evidence from longitudinal and experimental research is limited [5-10]. In the UK, and other countries, public health guidelines recommend that children should minimise the amount of time spent being sedentary for prolonged periods [11,12].

Research into the determinants of sedentary behaviour enables the identification of at-risk populations and modifiable factors that may be targeted within intervention programmes [13,14]. Contemporary thinking on the determinants of health behaviour advocates the application of an ecological framework to reflect the influence of factors operating at individual, social and environmental levels [15]. In children, the influence of home and familial characteristics on sedentary behaviour patterns has been a key area of research [16-19], particularly regarding the impact of electronic media (TV, computers, video games consoles) in the bedroom. Approximately two-thirds of children in the US and UK have a TV in their bedroom [20,21] and this has been associated with higher levels of screen-time (watching TV, using a computer), reduced sleep and increased risk of overweight [20,22-24]. The removal of such devices from the bedroom has been recommended [9,25-27]. However, few longitudinal studies have examined how electronic media ownership changes with age and whether changes in the bedroom media environment are associated with changes in sedentary behaviour patterns. Previous research examining the association between presence of a TV in the bedroom and total sedentary time has been conducted in pre-schoolers [28] or adolescents [29] or produced mixed findings [30]. Therefore, the aims of the current study were to (1) describe changes in bedroom media ownership over 4 years, from ages 9/10 to 13/14 years (3 waves of assessment) (2) examine the cross-sectional association of bedroom media with objectively measured sedentary time and self-reported screen-time at three time-points and (3) examine the association of changes in bedroom media ownership with changes in objectively measured sedentary time and self-reported screen-time.

## Methods

### Study design and setting

The Sport, Physical Activity, and Eating Behaviour: Environmental Determinants in Young People (SPEEDY) study is a population based longitudinal, cohort study investigating factors associated with physical activity, sedentary behaviour and dietary patterns in children from the county of Norfolk, UK [31]. Ethical approval was obtained from the University of East Anglia research ethics committee.

### Participants

Full details of participant recruitment and procedures for baseline data collection in SPEEDY have been reported previously [31]. From 227 eligible schools (those with >12 children in year 5), 157 were approached and 92 were recruited. At participating schools, all children in school year 5 and their parents (n = 3619) were sent an invitation pack. In total, 2064 children provided parental consent and were measured at baseline (57% of those invited).

### Data collection procedures

Participants were invited to participate on three separate occasions: baseline (T_0_; age 9/10y; April-July 2007), 1-year follow-up (T_1y_; age 10/11y; April-July 2008), and 4-year follow-up (T_4y_; age 13/14y; April-July 2011). Where possible, timing of follow-up assessments were matched to baseline. At baseline, trained research assistants visited schools to take physical measurements, administer child questionnaires, and fit accelerometers. Participants were requested to return the accelerometer one week later; Participation at T_0_ was prerequisite for recruitment to either of the subsequent waves of assessment. At T_1y_, study information sheets and consent forms were mailed to all 2064 initial participants [2]. Those who consented were mailed an accelerometer and a detailed instruction sheet. Participants were asked to wear the accelerometer for one week and to return it by mail, using an addressed, pre-paid envelope. At T_4y_, all participants with a valid home address from T_1y_ (n = 1964) were sent information sheets and consent forms. Through local administrative authorities, we ascertained the number of participants attending each secondary school in Norfolk, but our original consent did not allow us to trace individual participants. We presented the study in Year 9 assemblies at secondary schools attended by at least five original participants. Consent forms were returned to the study office by mail. Subsequent measurements were taken at school following similar procedures as at baseline. To increase recruitment, an extra invitation letter was sent home prior to the holiday (July 2011), resulting in an additional 62 participants being assessed by mail, following the same methodology as T_1y_.

### Objectively measured sedentary time

At each assessment, sedentary time was measured objectively using an Actigraph (GT1M; Pensacola, FL) accelerometer, [32,33] set to record at 5-second epochs. Children were instructed to wear the monitors during waking hours for 7 days and to remove them while bathing, showering and swimming. For quality control purposes, participants also received an accelerometer diary and instructed to indicate when the monitor was taken off and for what reason. Accelerometer data were analysed using a batch processing program (MAHUffe; http://www.mrc-epid.cam.ac.uk/research/resources/materials-transfer-disclaimer/physical-activity-downloads/). A count threshold of <100 counts per minute was used to define sedentary time [34,35]. Periods of ≥10 minutes of consecutive zero counts [2,36] and days with <500 minutes of recording between 6 am-11 pm were excluded [1,2]. A minimum of 3 days of valid accelerometer data, including 1 weekend day, was required for inclusion in the analysis. To optimise specificity between outcome and exposure measures, data collected during school hours (9 am-3 pm on weekdays) were excluded from the analysis.

### Self-reported screen-time

At T_0_ and T_4y_, leisure-time screen-time was assessed using a slightly modified version of a child self-report questionnaire, the Youth Physical Activity Questionnaire (YPAQ; [37]), which is based on the Children’s Leisure Activities Study Survey (CLASS; [38]). Separate items assessed time spent using a computer (including the internet; 1 week test-retest reliability ICC for CLASS items = 0.53) and watching TV (including video/DVD; 1 week test-retest reliability ICC = 0.93). Participants indicated on which of the previous 7 days they had engaged in these behaviours and the average duration of participation. Items referred to behaviours occurring outside of school hours but did not distinguish recreational from educational use. Weekly hours of screen-time was calculated by multiplying the frequency by the duration and summing the total for weekdays and weekend days. Time spent playing video games was not included in our screen-time calculation because the survey made no distinction between ‘active’ and ‘passive’ video games consoles.

### Bedroom media

The presence of a television or computer in the bedroom was self-reported by participants at T_0,_ T_1y_ and T_4y_. A combined bedroom media score (range 0-2) was calculated as the sum of the TV and computer items. Categorical variables indicating changes in bedroom TV and computer ownership (constantly absent, constantly present, T_0_ absent / T_4y_ present, T_0_ present / T_4y_ absent) and combined bedroom media items (stable, decrease, increase) from T_0_ to T_4y_ were derived.

### Covariates

All covariates were assessed at baseline. Age and sex were self-reported. Height and weight were measured by trained research assistants and used to calculate body mass index (BMI, kg/m^2^); weight status (normal weight/overweight) was determined using age and sex specific thresholds [39]. Postal code was used to determine urban/rural location of participants home [40]. Four density profiles were collapsed into a dichotomous variable (city / town and fringe: urban; hamlets and isolated dwellings / villages: rural). Because different markers of socioeconomic status (SES) may exhibit differential associations with sedentary behaviour [41], we derived a composite score (range 0-3) to better reflect the underlying SES construct; the score comprised parent-reported age at leaving full time education (≤16 years coded 0; >16 years coded 1), car ownership (no coded 0; yes coded 1), and house ownership (rental coded 0; own/buying coded 1). Participants were assigned to low (score 0/1), mid (score 2) or high (score 3) SES groups.

### Statistical analysis

Analyses were conducted using Stata (Stata, College Station, TX) in 2013. Baseline characteristics for those included and lost to follow-up were compared using *t* tests and *Χ*^2^ tests. Sex differences in sedentary time and screen-time were examined using *t* tests and Mann-Whitney tests respectively. Within time-point differences in bedroom media ownership according to sex, weight status, SES and urban/rural location were assessed using *Χ*^2^ tests. In the cross-sectional and longitudinal analyses, multi-level linear regression was used to examine the association of bedroom media with sedentary time and screen-time. Estimates of screen-time, but not change in screen-time, were non-normally distributed and therefore transformed (Box-Cox) prior to analysis. Regression models were adjusted for school-level clustering, sex, age, BMI, SES and urban/rural location. Interaction terms were used to assess potential effect modification by sex.

For descriptive purposes (Table 1), accelerometer derived sedentary time is presented as the proportion of wear time spent sedentary and weighted hours per week spent sedentary (5*weekday + 2*weekend). In cross-sectional analyses, the outcome was the proportion of wear time spent sedentary. In longitudinal analyses, the outcome was change in the proportion of wear time spent sedentary, calculated as follows: [(T_4y_ sedentary time/T_4y_ wear time) × 100]-[(T_0_ sedentary time/T_0_ wear time) × 100]. In both cases, outcome variables were continuous and normally distributed.

**Table 1:** Children’s objectively measured sedentary time and self-reported screen-time at 3 time-points (values are mean ± SD unless stated otherwise)

	**T**_**0 **_**(n = 1512)**	**T**_**1y **_**(n = 715)**	**T**_**4y **_**(n = 319)**
	**44.9% male, 10.3 (0.31)y**	**41.0% male, 11.2 (0.3)y**	**48.3% male, 14.3 (0.3)y**
**Accelerometer Sedentary time, % of wear time**			
**All**	62.2 ± 6.3	63.5 ± 6.3	70.2 ± 6.5
**Boys**	61.1 ± 6.7	62.4 ± 6.4	69.6 ± 7.2
**Girls**	63.1 ± 5.9**	64.3 ± 6.0**	70.8 ± 5.6
**Accelerometer Sedentary time, Hrs/wk**			
**All**	34.9 ± 5.3	35.6 ± 5.0	40.3 ± 5.3
**Boys**	34.6 ± 5.6	35.1 ± 5.3	40.3 ± 5.8
**Girls**	35.2 ± 5.1*	36.0 ± 4.8*	40.4 ± 4.8
	**T**_**0 **_**(n = 1745)**		**T**_**4y **_**(n = 373)**
	**44.0% male, 10.3 (0.3)y**		**45.0% male, 14.3 (0.3)y**
**Self-reported screen-time, Hrs/wk, median (IQR)**			
**All**	6.9 (2.9−14.8)	-	15.1 (8.5−26.0)
**Boys**	8.1 (3.3−16.6)	-	15.2 (8.9−25.5)
**Girls**	6.1 (2.6−13.2)**	-	15.0 (8.3−26.0)

T_0_, April-July 2007; T_1y_, April-July 2008; T_4y_, April-July 2011; y, years; SD, standard deviation; IQR, inter-quartile range.

*Within time-point difference between sexes (P < 0.05).

**Within time-point difference between sexes (P < 0.01).

## Results

Of the 2064 children that provided parental consent at baseline, valid accelerometer data was obtained for 1512 (T_0_, 73% of baseline participants), 715 (T_1y_, 35%), and 319 (T_4y_, 15%) participants respectively. Data on self-reported screen-time was provided by 1745 (T_0_, 85%) and 373 (T_4y_, 18%) participants respectively. With the exception of the sample providing screen-time data at T_0_, participants included in cross sectional analyses were of higher SES than those that were excluded (all p < 0.05). The sample providing valid accelerometer data at T_1y_ (p = 0.01) and T_4y_ (p < 0.01) also had lower BMI than participants who were excluded from analyses. Additionally, participants with valid accelerometer data at T_1y_ were more likely to be female (p = 0.01), younger (p = 0.05) and live in a rural location (p = 0.007) than participants without valid data. Data on objectively measured sedentary time and self-reported screen-time at T_0_, T_1y_ and T_4y_ are presented in Table 1.

**Figure 1:**
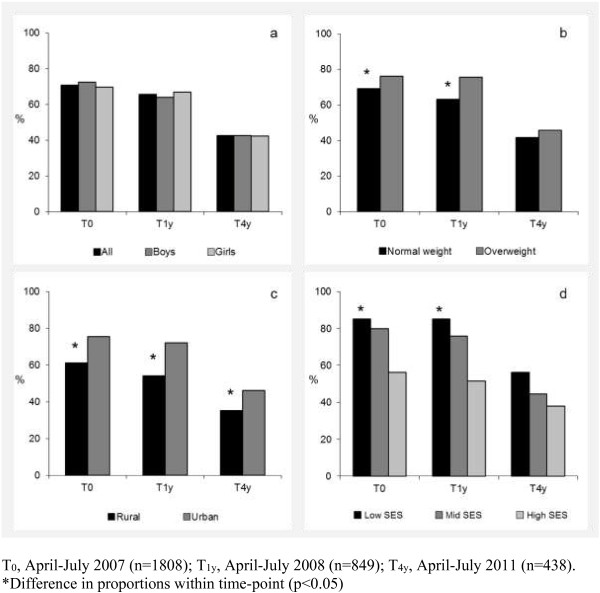
**Proportion of children with a TV in their bedroom, stratified by (a) sex, (b) weight status, (c) urban/rural location, (d) socioeconomic status.** T0, April-July 2007 (n=1808); T1y, April-July 2008 (n=849); T4y, April-July 2011 (n=438). *Difference in proportions within time-point (p<0.05).

**Figure 2:**
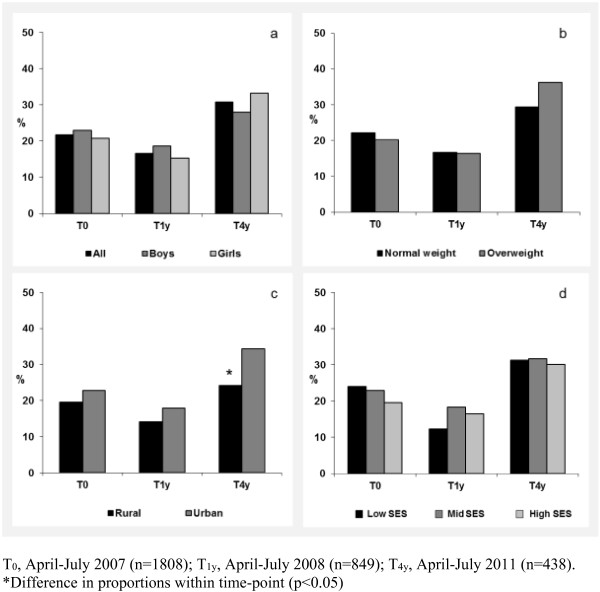
**Proportion of children with a computer in their bedroom, stratified by (a) sex, (b) weight status, (c) urban/rural location, (d) socioeconomic status.** T0, April-July 2007 (n=1808); T1y, April-July 2008 (n=849); T4y, April-July 2011 (n=438). *Difference in proportions within time-point (p<0.05).

**Figure 3:**
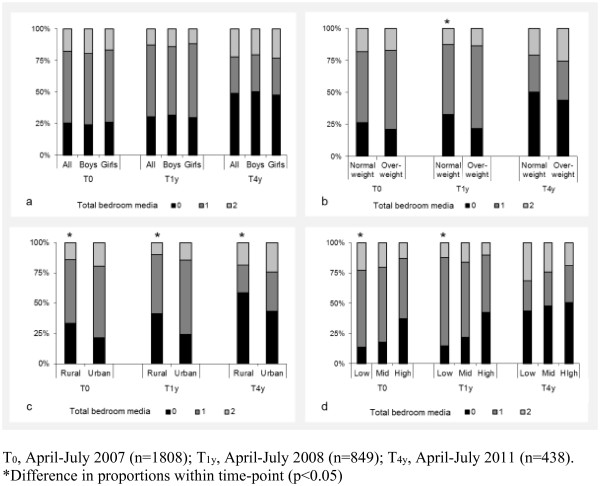
**Proportion of children with 0, 1, or 2 electronic media (TV/computer) in their bedroom, stratified by (a) sex, (b) weight status, (c) urban / rural location, (d) socioeconomic status.** T0, April-July 2007 (n=1808); T1y, April-July 2008 (n=849); T4y, April-July 2011 (n=438). *Difference in proportions within time-point (p<0.05).

The proportion of participants with a TV or computer in the bedroom is presented in Figures 1 and 2. Overall, 70.9% of participants had a TV in their bedroom at T_0_, declining to 42.5% at T_4y_. Relative to their respective reference groups, children who were overweight (T_0_ (p for χ^2^ = 0.009); T_1y_ (p = 0.002)), living in an urban area (T_0_ (p < 0.001); T_1y_ (p = <0.001); T_4y_ (p = 0.026)) or from families of low SES (T_0_ (p < 0.001); T_1y_ (p < 0.001)) were more likely to have a TV in the bedroom. The presence of a computer in the bedroom increased from 21.7% at T_0_ to 30.8% at T_4y_. At T_4y_ only, the proportion of participants with a computer in their bedroom was higher in children from urban compared to rural locations (p = 0.027). The proportion of participants with 0, 1, or 2 electronic media in the bedroom is presented in Figure 3. From T_0_ to T_4y_, the proportion of children with no electronic media in the bedroom increased from 25.2% to 48.9%. Concurrently, there was a small increase in the proportion of children with both a TV and computer in the bedroom (T_0_: 17.8%; T_4y_: 22.2%). Differences in combined bedroom media score were observed by weight status (T_1y_ p = 0.017), urban/rural location (T_0_ (p < 0.001); T_1y_ (p < 0.001); T_4y_ (p = 0.009)) and SES (T_0_ (p < 0.001); T_1y_ (p < 0.001)).

Cross-sectional and longitudinal associations of bedroom media with sedentary time and screen-time are presented in Tables 2 and 3 respectively. At T_0_ and T_1y_, having a TV in the bedroom and total bedroom media were negatively associated with objectively measured sedentary time. Having a computer in the bedroom and combined bedroom media score was positively associated with self-reported screen-time at T_0_ and T_4y_. Longitudinally, no significant associations were observed between changes in bedroom TV or computer ownership and change in objectively-measured sedentary time. Having a computer in the bedroom at T_0_ but not at T_4y_ was negatively associated with change in screen-time; participants in this group reported smaller increases in screen-time than those in the reference group. Tests for interaction by sex revealed that cross-sectional associations between bedroom media and objectively-measured sedentary time were typically stronger in girls, though few interactions attained significance (Additional file 1). No evidence of interaction with sex was observed for self-report outcomes or in longitudinal models.

**Table 2:** Cross-sectional association of bedroom media with objectively measured sedentary time and self-reported screen-time

	**T**_**0 **_**(n = 1512)**			**T**_**1y **_**(n = 715)**			**T**_**4y **_**(n = 319)**		
	**β (95% CI)**		***P***	**β (95% CI)**		***P***	**β (95% CI)**		***P***
Accelerometer sedentary time*									
**Television in bedroom**	−1.17	(−1.88, −0.46)	<0.01	−1.68	(−2.67, −0.70)	<0.01	0.21	(−1.23, 1.65)	0.77
**Computer in bedroom**	−0.49	(−1.25, 0.27)	0.21	0.29	(−0.91, 1.49)	0.64	1.25	(−0.32, 2.82)	0.12
**Combined bedroom media**	−0.76	(−1.26, −0.27)	<0.01	−0.79	(−1.51, −0.07)	0.03	0.51	(−0.40, 1.42)	0.27
	**T**_**0 **_**(n = 1745)**						**T**_**4y **_**(n = 373)**		
	**β (95% CI)**		***P***				**β (95% CI)**		***P***
**Self-reported screen-time**^**†**^									
**Television in bedroom**	0.06	(−0.07, 0.19)	0.35				0.22	(−0.02, 0.46)	0.07
**Computer in bedroom**	0.15	(0.02, 0.29)	0.02				0.35	(0.10, 0.60)	<0.01
**Combined bedroom media**	0.09	(0.01, 0.18)	0.03				0.20	(0.05, 0.34)	<0.01

Models adjusted for clustering in schools, sex, age, body mass index, socio-economic status and urban/rural location.

*Outcome variable: Weekly proportion of wear time spent sedentary; Beta coefficient (95% CI)*100.

^†^Outcome variable: Weekly hours of self-reported screen-time (Box-Cox transformed); Beta coefficient (95% CI).

**Table 3:** Longitudinal association of change in bedroom media with change in sedentary time and self-reported screen-time

		Sedentary time (n = 283)*				**Screen-time (n = 357)†**		
**Variable**	**n**	**β (95% CI)**		**P**	**n**	**β (95% CI)**		**P**
**Television**								
**Constantly absent**	90	Ref			112	Ref		
**Constantly present**	86	1.39	(−0.79, 3.57)	0.21	113	4.17	(0.18, 8.15)	0.04
**T**_**0 **_**absent – T**_**4y **_**present**	36	−0.57	(−3.25, 2.1)	0.68	38	3.59	(−1.89, 9.07)	0.20
**T**_**0 **_**present – T**_**4y **_**absent**	71	0.59	(−1.64, 2.82)	0.61	94	0.82	(−3.38, 5.01)	0.70
**Computer**								
**Constantly absent**	164	Ref			196	Ref		
**Constantly present**	23	0.49	(−2.56, 3.54)	0.75	32	1.96	(−3.51, 7.43)	0.48
**T**_**0 **_**absent – T**_**4y **_**present**	58	1.64	(−0.48, 3.75)	0.13	84	2.08	(−1.68, 5.83)	0.28
**T**_**0 **_**present – T**_**4y **_**absent**	38	0.53	(−2.97, 1.91)	0.67	45	−8.02	(−12.75, −3.29)	<0.01
**Combined bedroom media**								
**Stable**	126	Ref			155	Ref		
**Decrease**	80	−0.43	(−2.39, 1.53)	0.67	102	−1.88	(−5.62, 1.87)	0.33
**Increase**	77	0.49	(−1.51, 2.49)	0.63	100	2.57	(−1.2, 6.35)	0.18

Ref, reference group.

Models adjusted for clustering in schools, sex, age, body mass index, socio-economic status and urban/rural location.

*Outcome variable: Change in weekly proportion of wear time spent sedentary T_4y_-T_0_; Beta coefficient (95% CI)*100.

^†^Outcome variable: Change in weekly hours of self-reported screen-time T_4y_-T_0_; Beta coefficient (95% CI).

## Discussion

### Main findings

This study examined change in the electronic media environment of the bedroom over 4 years and the association of bedroom media with objectively measured sedentary time and self-reported screen-time. A complex pattern of findings emerged, likely reflecting both the true nature of the association between bedroom media and children’s sedentary behaviours and contemporary developments in electronic media ownership. Over 4 years, bedroom TV ownership declined substantially whilst the presence of a computer in the bedroom rose. In cross-sectional analyses, there was evidence of a positive association between bedroom media and children’s screen-time but associations with overall sedentary time were less clear. Longitudinal analysis indicated that removal of a computer from the bedroom may be a means of limiting the age-related increase in screen-time, but changes in the bedroom media environment were not associated with changes in overall sedentary time.

### Comparison with other evidence and implications

The observed increase in screen-time and overall sedentary time from T_0_ to T_4y_ is consistent with previous research indicating that sedentary behaviour increases as children age [1,42]. Reported screen-time in the current study was somewhat lower than has been reported previously in UK children; this may be due to temporal shifts in screen-time habits, contrasting sample characteristics or the application of different measurement tools [42] The extent to which the rise in overall sedentary time is attributable to increased screen-time or shifts in other behaviours, for example greater homework requirements or car use, remains unclear. More detailed characterisation of age-related changes in sedentary behaviour, that includes assessment of specific behaviours as well as overall sedentary time, will enable interventions to be developed and targeted more precisely.

The proportion of children with a TV in the bedroom decreased by approximately 30% points between T_0_ and T_4y,_ whilst the proportion of children with a computer in the bedroom increased by approximately 10% points over the same period. Recent data from Ofcom (the independent regulator for the UK communications industries) also indicates a decline in the presence of TVs and a rise in the availability of the internet in children’s bedrooms between 2007–2010, though the magnitude of change was smaller than that seen in the current study [21]. Findings may reflect age related changes in media preferences [21] but also broader societal patterns in electronic media ownership over the study period. In a 2011 survey, computers (including ‘tablet’ computers), e-book readers and mobile phones were listed above television sets as items that US children and adolescents had most interest in buying within the next 6-months [43]. Television viewing in the traditional sense (watching live or time shifted content on a television set delivered by broadcast signal or paid TV subscription) appears to be declining, as consumers increasingly utilise alternative devices (e.g. computers, games consoles) to access video content [44,45]. The evolution and uptake of electronic media, both portable and home-based, frequently outpaces the efforts of researchers to document trends and examine its impact upon behaviour and health. Going forward, researchers should recognise the multitude of platforms through which children may access audio-visual content and acknowledge the increasing portability and multifunctionality of new devices.

The proportion of participants with a TV or computer in the bedroom differed by socioeconomic status and by urban/rural location. Previous studies have reported that having a TV in the bedroom is typically more common amongst low SES families, whilst having a computer or internet access in the bedroom is more prevalent in higher SES groups [21,46]. The use of different markers to indicate SES, however, limits comparability between studies. An urban/rural divide in internet usage and connection speeds has been reported previously in the UK [47,48] but we are unaware of any existing studies that have examined urban/rural differences in the bedroom media environment.

In this study, combined bedroom media score was positively associated with screen-time at T_0_ and T_4y_, consistent with previous research [18,49]. Interestingly, the positive association observed in this study appears to be driven predominantly by the presence of a computer in the bedroom, which was individually associated with screen-time at T_0_ and T_4y_. Considered alongside our finding that the presence of a computer in the bedroom increased from T_0_ to T_4y_, these data suggest that researchers must acknowledge emerging trends in electronic media ownership and avoid an overly restrictive focus solely upon TV [27]. In so doing, new instruments may be required to capture the diversity of electronic media used by young people. It may also be valuable to concurrently ascertain context of behaviour and postural allocation, as it may no longer be appropriate to infer posture on the basis of reported behaviour.

Unexpectedly, having a TV in the bedroom and combined bedroom media score were negatively associated with objectively measured sedentary time at T_0_ and T_1y_. There was some evidence that the negative association of bedroom media with sedentary time was stronger in girls than boys but interaction tests were mostly borderline significant. Previous studies have predominantly shown no association between having a TV in the bedroom and accelerometer-determined sedentary time and no evidence for interaction with sex [28,30]. Given the increasing use of accelerometry to assess sedentary time in epidemiological studies, further research on this question is likely and may provide some clarity. It is also of interest that cross-sectional analyses at T_4y_ identified (non-significant) positive associations between bedroom media and sedentary time. It is unclear whether this apparent switch in the direction of the association is arbitrary (associations *were* non-significant at T_1y_ and T_4y_), an artefact of study design, or reflects a changing pattern of influence as children age. Further studies exploring this potential interaction with age are required.

In longitudinal analyses, a smaller increase in screen-time was observed in participants that reported having a computer in the bedroom at T_0_ but not at T_4y_. In most models, the associations between a change in bedroom media and change in sedentary time or screen-time were in the anticipated direction but did not attain statistical significance. Participant attrition may have resulted in reduced statistical power for these analyses. Numerous studies have advocated the removal of electronic media from children’s bedrooms as a means of limiting screen-time, [9,25-27] but experimental research exploring the efficacy of this approach is lacking. Findings of the current study provide some support for this strategy. However, important contextual information regarding the circumstances in which computers were removed from participants’ bedrooms is lacking in our analysis. Moreover, qualitative work has indicated that children and parents may be resistant to the idea of removing electronic media from the bedroom once they have been installed [50]. Trials exploring the influence of removing electronic media from children’s bedrooms should include process evaluations to understand the acceptability of this strategy for children and parents.

In the current study, we are not able to ascertain whether observed changes in the bedroom media environment were attributable to age-related changes in media preferences or broader societal changes in electronic media use over the study period. Uptake of information technology and communications media has grown rapidly in recent years [20,21]. Devices such as tablet computers and mobile phones are highly valued by adolescents and are an increasingly important means by which young people accumulate screen-time [20,21,43]. They are also portable and multifunctional, enabling the user to perform multiple tasks (e.g. gaming, using the internet, watching TV) simultaneously and without being tied to a specific location. Researchers need to recognise the broad range of electronic media used by young people, including the extent to which users may perform multiple tasks simultaneously, and be more explicit in gathering data on the contexts in which behaviours occur, rather than inferring context from the location of a particular device [26].

### Strengths and limitations

The strengths of this study include its longitudinal design and the collection of objective and self-report data on sedentary behaviour in a large population-based sample of children. Repeated assessments on a single cohort enabled us to identify changes in behaviour and the bedroom media environment over time. Regression models were adjusted for a number of potentially confounding factors and interactions with sex were explored. A limitation is that video game consoles were not included in our assessment of the bedroom media environment or in our screen-time outcome. This is because we were unable to differentiate between active and passive games consoles in baseline assessments. In addition, we did not assess ownership of tablet computers or distinguish between desktop or laptop computers in our questionnaire. We addressed the potential limitation of differences in accelerometer wear between waves of assessment by deriving outcomes that were relative to recorded wear time at each time point. The response rate at T_4y_ was low and participants with higher BMI or from lower SES families were less likely to provide outcome data at T_1y_ and T_4y_, limiting generalizability of findings. Where appropriate, we conducted sensitivity analyses to examine the potential impact of selection bias by limiting the analytical sample to participants that provided complete data at all time points. In all cases, the direction and magnitude of associations were minimally affected (data not shown). Lastly, information on the covariates SES, BMI and urban/rural location of the home was collected at baseline only; it is possible that these factors may have changed over time, possibly resulting in misclassification.

## Conclusion

In this study we found that there were notable changes in the electronic media environment of children’s bedrooms over 4 years, and that the presence of bedroom media was more consistently associated with children’s screen-time behaviour than overall sedentary time. In light of the rapid and continuing uptake of information and communications technology by young people, further research examining the context and content of electronic media use, and its impact upon behaviour and health, is essential.

## Competing interests

The authors declare that they have no competing interests.

## Authors’ contributions

AJA conceived the analysis, performed data analysis and drafted the manuscript. KC contributed to study design, data acquisition and analysis, and critically reviewed the manuscript. EMFS contributed to study design, data acquisition and analysis, and critically reviewed the manuscript. All authors read and approved the final manuscript.

## Supplementary Material

Additional file 1 Cross-sectional association of bedroom media with objectively measured sedentary time, stratified by sex. Click here for file
