# Supplementary material for: Bedroom media, sedentary time and screen-time in children: a longitudinal analysis
Source: Int J Behav Nutr Phys Act. 2013 Dec 17;10:137. doi: 10.1186/1479-5868-10-137 (PMC3895703; doi:10.1186/1479-5868-10-137)
Supplement: Additional file 1 — Cross-sectional association of bedroom media with objectively measured sedentary time, stratified by sex. [file 1479-5868-10-137-S1.doc]

Supplementary table

Table 4. Cross-sectional association of bedroom media with objectively measured sedentary time, stratified by sex

|  |  | **T_0_** | | |  | **T_1y_** | | |  | **T_4y_** | | |
| --- | --- | --- | --- | --- | --- | --- | --- | --- | --- | --- | --- | --- |
| **Variable** |  | **β (95% CI)** | | ***P*** |  | **β (95% CI)** | | ***P*** |  | **β (95% CI)** | | ***P*** |
|  |  |  |  |  |  |  |  |  |  |  |  |  |
| **TV in bedroom**  **Boys (n=679)**  **Girls (n=833)**  **P for interaction** |  | -0.63  -1.72  - | (-1.77, 0.52)  (-2.62, -0.83)  - | 0.28  <0.01  0.07 |  | -1.02  -2.04 | (-2.62, 0.57)  (-3.28, -0.81) | 0.21  <0.01  0.24 |  | 0.18  0.39 | (-2.08, 2.44)  (-1.39, 2.18) | 0.88  0.67  0.98 |
| **Computer in bedroom**  **Boys (n=293)**  **Girls (n=422)**  **P for interaction** |  | 0.06  -0.94 | (-1.14, 1.26)  (-1.91, 0.03) | 0.92  0.06  0.13 |  | 1.10  -0.35 | (-0.78, 2.99)  (-1.91, 1.22) | 0.25  0.67  0.23 |  | -0.17  2.41 | (-2.85, 2.51)  (0.59, 4.23) | 0.90  0.01  0.11 |
| **Combined bedroom media**  **Boys (n=154)**  **Girls (n=165)**  **P for interaction** |  | -0.26  -1.24 | (-1.04, 0.52)  (-1.87, -0.62) | 0.51  <0.01  0.02 |  | -0.12  -1.26 | (-1.26, 1.03)  (-2.18, -0.33) | 0.84  <0.01  0.09 |  | 0.03  1.03 | (-1.46, 1.51)  (-0.08, 2.14) | 0.97  0.07  0.33 |

T_0_, April-July 2007; T_1y_, April-July 2008; T_4y_, April-July 2011. Models adjusted for clustering in schools, age, body mass index, socio-economic status and urban/rural location

Outcome variable: Weekly proportion of wear time spent sedentary (non-school); Beta coefficient (95% CI)*100.
